# The medial habenula as a regulator of anxiety in adult zebrafish

**DOI:** 10.3389/fncir.2013.00099

**Published:** 2013-05-27

**Authors:** Ajay S. Mathuru, Suresh Jesuthasan

**Affiliations:** ^1^Neural Circuitry and Behavior Laboratory, Institute of Molecular and Cell BiologySingapore, Singapore; ^2^Neuroscience and Behavioral Disorders Program, Duke-NUS Graduate Medical SchoolSingapore, Singapore; ^3^Department of Physiology, Yong Loo Lin School of Medicine, National University of SingaporeSingapore, Singapore

The habenula consists of a set of nuclei located in the epithalamus. It regulates the release of multiple neuromodulators including serotonin and dopamine, and consists of two major subdivisions—medial and lateral. In all vertebrates, the medial habenula projects to the interpeduncular nucleus (IPN), a midline structure with poorly defined functions (Morley, 1986). Both the medial habenula and the IPN are rich in nicotinic receptors (nAChR). Activity in this pathway, triggered by opioids and nicotine, leads to a rise in dopamine in the nucleus accumbens (Glick et al., 2006; McCallum et al., 2012) and thus underlies the rewarding aspect of substance abuse. Strong activation of nicotinic receptors in the medial habenula or IPN, however, is sufficient to mediate the aversion to high concentration of nicotine (Fowler et al., 2011; Frahm et al., 2011). In contrast, absence of activity in this pathway is critical for the effects of withdrawal (Salas et al., 2009; Baldwin et al., 2011). Hence, depending on the level of activity, the medial habenula-IPN pathway can trigger reward, aversion or the physical and emotional changes that are characteristic of withdrawal.

The medial habenula regulates the expression of fear in zebrafish. Following silencing with tetanus toxin or lesioning with nitroreductase (Agetsuma et al., 2010; Lee et al., 2010), enhanced freezing was seen in aversive conditioning paradigms in both adult and larvae. The freezing response in habenula-lesioned fish was experience-dependent. In the case of adults, both control and lesioned fish froze when first exposed to an electric shock paired with a red light (the conditioning stimulus, CS) in a Pavlovian conditioning paradigm. As more shocks were delivered, freezing decreased in control fish, but not in habenula-lesioned fish (Agetsuma et al., 2010). The experiment with larval fish involved instrumental conditioning: fish had to swim away from the side of the tank containing a red light, which had been paired with a mild electric shock. In this case, freezing appeared gradually in lesioned fish in the later half of the conditioning session, but never in control fish (Lee et al., 2010).

One interpretation that has been suggested for these observations is that silencing the medial habenula biases fish to a passive coping strategy of freezing, due to modulation in the activity of the downstream griseum centrale [homologous to periaqueductal gray (PAG) in mammals] and nucleus incertus (Okamoto et al., 2012). As different regions of the PAG have been implicated in selection of different actions for coping against stress, a role for the medial habenula in such selection via PAG is a distinct possibility. An additional possibility is that medial habenula silencing increases anxiety in animals.

**Figure 1:**
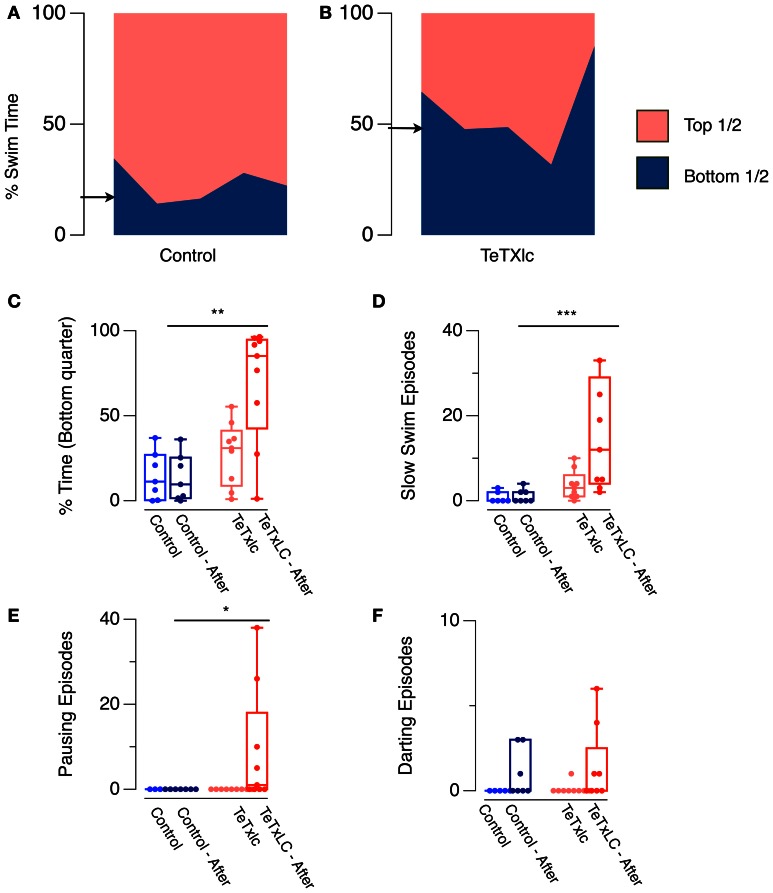
**(A,B)** Novel tank assay. Area in each color shows percentage time spent in either half of a novel tank in one minute by five Control fish and five TeTXlc expressing fish. Arrows indicate median swim time in the bottom half for controls (20.4 ± 3.7%) and TeTXlc fish (48.6 ± 9%). **(C–F)** Response to dilute alarm substance and an overhead shadow. **(C)** Time spent in the bottom quarter of a tank, and median number of **(D)** slow swimming, **(E)** pauses, and **(F)** darts. Non-parametric, Mann–Whitney *U*-test was used to compare behavior of control and TeTXlc fish following exposure to the stimuli. (Mann–Whitney *U*-test, ^*^p < 0.05, ^**^p < 0.01, ^***^p < 0.001). Zebrafish expressing TeTXlc-CFP in the habenula were generated by crossing GAL4^s1019t^ with UAS:TeTXlc-CFP, and selected on the basis of CFP fluorescence in the brain. For the novel tank assay, fish were transferred into a glass tank measuring 20 × 12 × 5 cm—L × H × W containing 1 L of water and observed for 1 min. Digital videos were recorded as described before (Mathuru et al., 2012). Fish position in the tank was tracked and analyzed automatically using “track objects” algorithm in MetaMorph. 6.3 and custom written macros for Excel. Alarm substance was prepared as described before (Mathuru et al., 2012). This was introduced into the tank and a shadow, created by passing a hand under the overhead light, was presented after 2 min. Darting was defined as episodes during which the swimming speed exceeded baseline speed by more than 8 SD, slow swim episodes were defined as episodes greater than one second in duration during which the swim speed never exceeded half the mean baseline speed, while one second of immobility (speed less than 3 mm/s) was considered as a pause episode.

A consequence of elevated generalized anxiety in an animal is predisposition toward larger fear responses (Davis et al., 2010). The swimming behavior of an isolated fish when introduced into a novel tank can be used as a measure of baseline generalized anxiety (Levin et al., 2007): anxious fish spend more time near the bottom in a novel tank. Therefore, by using this assay, it is possible to quantify if adult fish where the medial habenula has been silenced display a similar, a lower, or a higher level of baseline anxiety when compared to control fish. We compared the behavior of adult fish that express the light chain of tetanus toxin (TeTXlc) in the medial habenula (Lee et al., 2010), under the control of the GAL4^s1019t^ driver (Scott et al., 2007), with fish that did not express this transgene. TeTXlc expressing fish spent more time in the bottom half of the tank compared to controls (Figures 1A,B), consistent with the possibility that these fish may indeed have a higher baseline generalized anxiety.

Mild stressors can elicit disproportionate reactions in anxious animals. In rats, for example, strong illumination, which is anxiogenic, causes an abnormally large startle to a tone (Walker and Davis, 1997). We examined the response of TeTXlc-expressing fish to naturalistic stimuli that are expected to be mildly stressful. Low concentration of the alarm substance (Jesuthasan and Mathuru, 2008) normally triggers a response that naïve, control fish recover from within seconds (0.1 unit; Mathuru et al., 2012). We followed this with an overhead shadow, which is thought to mimic the threat of a predator (Luca and Gerlai, 2012). These stimuli provide an opportunity to examine the role of medial habenula when the subject is challenged with stimuli that mimic complex natural stressors as opposed to its role in conditioning experiments that use unnatural repetitive stimuli. In such conditions, control fish displayed behavior indicative of mild fear—marginal increase in episodes of darting [Wilcoxon Signed Rank Test (*n* = 7), *p* = 0.1; Figure 1F], while other measures remained unchanged. TeTxlc-expressing fish, in contrast, spent more time spent in the bottom quarter of the tank (Mann–Whitney *U*-test, n1, n2, 7, 9, *U* = 7, *p* = 0.005; Figure 1C), displayed more episodes of slow swimming (Mann–Whitney *U*-test, n1, n2, 7, 9, *U* = 3, *p* = 0.001; Figure 1D) and pauses (Mann–Whitney *U*-test, n1, n2, 7, 9, *U* = 14, *p* = 0.021; Figure 1E). Darting episodes were comparable to controls (MW n1, n2, 7, 9, *U* = 17.5, *p* = 0.435; Figure 1F). The response of TeTxlc-expressing fish resembled that of naïve fish that exhibit intense innate fear upon exposure to a high concentration of the alarm substance (1 unit; Mathuru et al., 2012). In other words, TeTxlc-expressing fish show a disproportionate response when presented with multiple mildly stressful stimuli. Notably, TeTxlc-expressing fish display both active and passive responses, suggesting that there is an increase in the intensity of fear expression rather than a bias toward a particular type of action.

By examining responses to a novel environment, as well as to the alarm substance along with an overhead shadow, i.e., conditions where experience is absent, the behavioral responses of fish with dysfunctional medial habenula appear to be consistent with that of animals in a state of elevated baseline anxiety. Behavioral deficits are manifest primarily in the presence of stressors, similar to the effects of nicotine withdrawal in rats (Jonkman et al., 2008).

The mechanism by which the habenula regulates a response to stress, both experience dependent and independent is likely to be complex, given the multiplicity of targets and modulators in the medial habenula-IPN pathway, which include somatostatin (Morley et al., 1985), met-Enkephalin, and substance P (Hamill et al., 1984). It is possible that regulation of serotonergic neurons in the raphe by the IPN also plays a role in modulating the response to aversive stimuli. Another intriguing hypothesis that has been suggested recently is that the absence of nicotine in addicts causes an increase in activity in the lateral habenula, which then leads to a decrease in dopamine release from the ventral tegmental area (VTA), thereby causing symptoms associated with withdrawal that include increased anxiety (Baldwin et al., 2011).

Is it possible then that silencing the medial habenula has a similar outcome, i.e., leads to an increase of activity in the lateral habenula? Consistent with the notion that the medial habenula can regulate lateral habenula outputs, projections from the medial to the lateral habenula have been detected in the rat (Kim and Chang, 2005), although it is not known if these projections are inhibitory. As the lateral habenula is able to use errors in prediction (based on experience) to trigger a change of state (Matsumoto and Hikosaka, 2007; Hong and Hikosaka, 2008), this region may also be involved in mediating the experience-dependent deficits of silencing the medial habenula. If activity in an inhibitory pathway from the medial habenula to the lateral habenula is reduced, the overall inhibitory tone on lateral habenula will also be reduced. In this scenario, transmission via the lateral habenula would be increased, and the outcome would be similar to that seen in rats where such an increase is associated with a predisposition for helpless behavior (Li et al., 2011), which includes increased freezing.

Thus, one step to understand both nicotine withdrawal and anxiety will be to characterize signaling between medial and lateral subnuclei of the habenula, in addition to examining downstream circuits. These subnuclei have remained in close proximity throughout vertebrate evolution, and it is possible that intra-habenula signaling modulates output, eventually effecting anxiety and motivated behaviors in animals.
